# Expectation-Maximization Model for Substitution of Missing Values Characterizing Greenness of Organic Solvents

**DOI:** 10.3390/molecules23061292

**Published:** 2018-05-28

**Authors:** Gabriela Łuczyńska, Francisco Pena-Pereira, Marek Tobiszewski, Jacek Namieśnik

**Affiliations:** 1 Division of Applied Mathematics and Probability, Institute of Mathematics, Faculty of Mathematics, University of Gdansk, 8 J. Bażyńskiego St., 80-309 Gdańsk, Poland; gabluczy@student.pg.edu.pl; 2 Department of Nonlinear Analysis and Statistics, Faculty of Applied Mathematics, Gdańsk University of Technology (GUT), 11/12 G. Narutowicza St., 80-233 Gdańsk, Poland; 3 Department of Analytical and Food Chemistry, Faculty of Chemistry, University of Vigo, Campus As Lagoas-Marcosende s/n, 36310 Vigo, Spain; fjpena@uvigo.es; 4 Department of Analytical Chemistry, Chemical Faculty, Gdańsk University of Technology (GUT), 11/12 G. Narutowicza St., 80-233 Gdańsk, Poland; marektobiszewski@wp.pl

**Keywords:** E-M algorithm, green analytical chemistry, missing data prediction, solvents, sustainability assessment

## Abstract

Organic solvents are ubiquitous in chemical laboratories and the Green Chemistry trend forces their detailed assessments in terms of greenness. Unfortunately, some of them are not fully characterized, especially in terms of toxicological endpoints that are time consuming and expensive to be determined. Missing values in the datasets are serious obstacles, as they prevent the full greenness characterization of chemicals. A featured method to deal with this problem is the application of Expectation-Maximization algorithm. In this study, the dataset consists of 155 solvents that are characterized by 13 variables is treated with Expectation-Maximization algorithm to predict missing data for toxicological endpoints, bioavailability, and biodegradability data. The approach may be particularly useful for substitution of missing values of environmental, health, and safety parameters of new solvents. The presented approach has high potential to deal with missing values, while assessing environmental, health, and safety parameters of other chemicals.

## 1. Introduction

Green Chemistry has been defined as the “design of chemical products and processes to reduce or eliminate the use and generation of hazardous substances” [1]. With this aim, Anastas and Warner, introduced, in 1998, the twelve principles of Green Chemistry that charted a path towards sustainability in chemical processes [1]. Several principles of Green Chemistry point out the need to eliminate or replace solvents by less harmful alternatives [2]. Particularly, the 5th principle of Green Chemistry specifically recommends the use of innocuous solvents when avoiding the use of solvents is not possible. The employment of harmful solvents is also indirectly discouraged, as can be deduced from additional principles, such as waste prevention (1st principle) and the prevention or minimization of potential chemical accidents (12th principle) that are associated to their use. Furthermore, advances toward the design of safer chemicals (4th principle) that at the end of their function can be transformed into innocuous non-persistent products (10th principle), and importantly, renewable feedstocks, rather than depleting non-renewable resources (7th principle), are highly recommended [3,4].

Organic solvents are increasingly used in scientific and technological activities, with an estimated worldwide consumption of roughly 30 million metric tons per year [5]. Apart from the steady increase of solvent consumption in the last years, especially worrisome is the fact that certain solvents of very high concern are still being widely used. Thus, the implementation of solventless process is strongly advisable from the point of view of Green Chemistry. While remarkable efforts have been made in certain areas toward the implementation of solventless processes (e.g., solventless sample preparation approaches [6] and greener reactions under solvent free conditions [7]), these strategies are, in general terms, still far from reaching the desired level of implementation. Alternatively, the minimization of solvent consumption and/or the replacement of hazardous solvents by cleaner alternatives are highly recommended strategies to reduce the risks that are associated to solvent usage [8,9]. In this vein, a number of solvent selection guides have been reported in the literature for a convenient selection of alternatives to harmful solvents [10,11,12,13,14,15,16,17,18]. However, the lack of relevant data, such as physicochemical properties and environmental impact, might hinder their implementation in scientific and technological processes [19]. The problem of missing data in solvents assessments is managed by default, substitution with value for nearest neighbor (homologue), and substitution with mean value for the entire chemical class [20].

Expectation-Maximization (E-M) algorithm [21] was developed in the 1970‘s and it is widely applied in different branches of sciences as the tool for the substitution of missing values [22]. It is applied to deal with missing values for the characterization of patients to predict breast cancer recurrence [23]. The algorithm is used to predict missing values in genetic arrays [24]. Application in chemistry include the prediction of missing data in environmental monitoring [25] or to construct regression models in the case of missing data in the raw dataset [26]. Other applications of E-M algorithm for predictions in the chemistry related field include the prediction of biomarkers essentiality [27] or the prediction of peptide bounding [28].

The aim of the study is to substitute missing values in the dataset characterizing organic solvents with the application of E-M algorithm, and to find relations between the characteristics from the estimated distribution. Toxicity, biodegradability, and bioavailability parameters are predicted with E-M algorithm.

## 2. Materials and Methods

### 2.1. Dataset

The dataset consists of 155 solvents that are described by 13 variables. The values of variables are extracted mainly from the Handbook of Physical-Chemical Properties and Environmental Fate for Organic Chemicals [29] and from material safety data sheets of solvents. Physicochemical properties include melting point, boiling point, vapor pressure, density, water solubility, Henry’s law constant, logarithms of octanol-water partitioning coefficient, and logarithm of octanol-air partitioning coefficient. Also, toxicity towards rodents when being administered orally (Oral LD_50_), toxicity towards rodents via inhalation exposure pathway (Inhalation LC_50_), toxicity towards fish (Fish LC_50_), half-life time needed for biodegradation, and logarithm of bioconcentration factors were taken as variables for analysis.

Solvents included in the dataset are compounds of different chemical classes–from hydrocarbons, terpenes, chlorinated solvents to alcohols, ketones, ethers, and esters and carboxylic acids. 85 out of the 155 solvents were fully characterized in terms of abovementioned variables, whereas the dataset contained at least one gap in case of the remaining solvents.

### 2.2. E-M Model

To complete the data we use E-M algorithm. This algorithm consists of two steps: an Expectation step or the E-step and a Maximization step or the M-step.

We observe a data $y=\left(y_{1},\ldots ,y_{n}\right)$, where $y_{i}$ are realizations of a random vector Y, i=1,…,n. Let Y has the probability distribution function depending on a vector of unknown parameters Ψ.

Let X be a k dimensional random vector corresponding to a complete-data $x=\left(x_{1},\ldots ,x_{n}\right)$, where $x_{i}$ are realizations of X, i=1,…,n. We consider the case where the vector X has multivariate normal distribution, which means that Ψ=(μ,Σ), where μ is a vector of means and Σ is a covariance matrix.

Suppose that there are G groups with distinct missing patterns. Then, the observed-data log likelihood can be expressed as

(1) $$\log L\left(\Psi \right)=\Sigma_{g=1}^{G}\log L_{g}\left(\Psi \right).$$

The likelihood function for gth group formed from the observed data $y_{g}=\left(y_{1g},\ldots ,y_{n_{g}g}\right)$ is, discarding a proportionality constant, given by

(2) $$L_{g}\left(\Psi \right)={\left|\Sigma_{g}\right|}^{-\frac{n_{g}}{2}}\exp\left\{-\frac{1}{2}\Sigma_{i=1}^{n_{g}}{\left(y_{ig}-\mu_{g}\right)}^{T}\Sigma_{g}^{-1}\left(y_{ig}-\mu_{g}\right)\right\}$$

An estimate $\hat{\Psi }$ of Ψ can be obtained by solving the log likelihood equation

(3) $$\frac{\partial \log L\left(\Psi \right)}{\partial \Psi }=0$$

The E-M algorithm approaches the problem of solving the incomplete-data log likelihood Equation (3) indirectly by proceeding iteratively in terms of complete-data log likelihood function $\log L_{c}\left(\Psi \right)$, where

(4) $$L_{c}\left(\Psi \right)={\left|{\left(2\pi \right)}^{k}\Sigma \right|}^{-\frac{n}{2}}\exp\left\{-\frac{1}{2}\Sigma_{i=1}^{n}{\left(x_{i}-\mu \right)}^{T}\Sigma^{-1}\left(x_{i}-\mu \right)\right\}$$

The E-step calculates the conditional expectation of the complete-data log likelihood, $E_{\Psi^{\left(0\right)}}\left(\log L_{c}\left(\Psi \right)|y\right),$ given the observed data and the parameter estimates. Then, the M-step finds the parameter estimates to maximize the complete-data log likelihood from the E-step.

The steps are carried out until the value of

(5) $$L\left(\Psi^{\left(k+1\right)}\right)-L\left(\Psi^{\left(k\right)}\right)$$

is smaller than arbitrarily amount in case of convergence of the sequence of likelihood values ${\left(L\left(\Psi^{\left(k\right)}\right)\right)}_{k}$. The extended description of this procedure can be found in Supplementary Material. More details for normal distribution can be found in [30].

### 2.3. Dataset Preparation

We consider 155 solvents that are described by 13 attributes, which are listed in Table 1. Some chemical compounds do not have the value for the parameter “Inhalation LC_50_”, because they are not volatile, so there is no possibility to be intoxicated via inhalation. We decided to give them values 5001. This is the expert judgment caused by the threshold of danger to the environment. According to Globally Harmonized System of Classification and Labelling of Chemicals, the values above 5000 ppm are not characterized as “harmful if inhaled” [31].

## 3. Results and Discussion

### 3.1. Basic Statistics

Table 1 shows the basic statistics of investigated dataset, including the number of missing data (N missing). For boiling point, density, water solubility, vapor pressure, and log K_OW_, all of the values are available. The biggest problems with data availability are in case of Oral LD_50_, inhalation LC_50_, fish LC_50_, and BOD t_1/2_ (biodegradation half-life), as they are characterized by a big fraction of missing values.

### 3.2. Predictions with Bayesian Model

#### Application of E-M Algorithm

To complete the data set, we use the programming language SAS 4GL. There is a proper procedure in SAS, called PROC MI, which performs the E-M algorithm by function EM. The extensive description of this procedure can be found in [32].

We assume that the random vector *X* corresponding to a complete-data vector *x* has the multivariate normal distribution, *X* ~*N*(*µ*,*Σ*) where *µ* is a vector of means and *Σ* is a covariance matrix. To approach that assumption, we use a logarithmic transformation of the properties Henry’s law constant, Oral LD_50_, Inhalation LC_50_, fish LC_50_, and BOD t_1/2_.

In this case, we have to find a parameter *Ψ =* (*µ*, *Σ*) where *µ* is a vector of means and *Σ* is a covariance matrix of the unknown distribution. The initial estimates *Ψ*^(0)^ are the means and the standard deviations from available cases. The correlations are set to zero.

On the prepared set, we carry out the E-M algorithm in SAS. To satisfy the convergence requirement the difference (5) has to be smaller than 0.0001. The method converges after 69 iterations. It means that

*L*(*Ψ*^(69)^) *− L*(*Ψ*^(68)^) *<* 0.0001 (6)

due to (5). Thereby, we received the completion of the data set. The most important thing is that we received full information about the set by finding the distribution of the data, *N*(*µ*,*Σ*). The E-M algorithm evaluated the parameters *µ* and *Σ.* Despite the Mardia’s kurtosis test has not shown that the data has a multivariate normal distribution we think that such a model gives a good approximation of relations. We will find these relations using principal component analysis (PCA) [33]. Table 2 shows the obtained results of first three principal components.

The first three components explain 65.9% of variability of the raw dataset. Fourth principal component explained 8.4% of variability, and it was decided not to include it in the assessment result. The first component consists of melting point, boiling point, vapor pressure, and log K_OA_, and it explains 26.2% of the initial variability. This component can be identified as responsible for the characterization of solvents in terms of their basic physicochemical properties, especially volatility. The second component is loaded with Henry’s law constant, log Kow, and log BCF, and it explains 24.4% of variability. This relation can be explained by the polarity of solvents. All three variables are related to interphase transfer and the ability to be transferred out of water. Apart from these three variables, weaker negative loading (−0.3774) is observed for water solubility, which additionally supports the polarity related origin of this group. The third component is formed by inhalation and oral toxicities and the negative loading of density. It carries 15.3% of initial dataset variability. It can be defined as toxicity relation and the presence of density in this group is due to the fact that more toxic solvents are usually more dense (i.e., chlorinated solvents).

The prediction of missing values are presented in Table 3—shaded are modeled with the E-M algorithm, whereas non-shaded are input data. Algorithm allows to substitute missing values for alkyl glycerol esters (numbers 15–29 in the Table 3), bio-based solvents originating from biodiesel production. Glycerol is formed as a byproduct during biodiesel production, and it is a platform molecule for the synthesis of its alkylated ester derivatives [34]. Because they originate from renewable resource, undergo biodegradation, and are low cost, they are potentially attractive from a Green Chemistry point of view. However, they are not fully characterized in terms of their toxicology or environmental fate related properties. The means of completed values are of 2017 mg kg^−1^ LD_50_ by oral administration (mean for solvents with available data—3667 mg kg^−1^), 13945 ppm of LC_50_ by inhalation (mean for solvents with available data—4658 ppm), 158 mg dm^−3^ of LC_50_ towards fish (mean for solvents with available data—970 mg dm^−3^), 1.96 days of biodegradability half-lifes (mean for solvents with available data—55 days), and 0.46 of logarithm of bioconcentration factors (mean for solvents with available data—1.15). According to Globally Harmonized System of Classification and Labelling of Chemicals, oral toxicity of >2000 mg kg^−1^ and inhalation toxicity of >5000 ppm indicate that they are chemicals of low acute toxicity. The predicted results show that alkyl glycerol esters may be toxic (especially towards fish) and they should be characterized in this manner to confirm their green status. Other predicted that missing values show that alkyl glycerol esters are biodegradable and they do not undergo bioaccumulation.

Esters (mainly methyl or isopropyl esters of fatty acids) are characterized by low completed inhalation toxicity, rather low oral toxicity, but they some have low values (at the level of single mg dm^−3^) of toxicity towards fish, which suggest they are potential threats to aquatic life. Their completed values suggest that they are biodegradable, with half-lifes at the level of few to 20 days. Gamma valerolactone is considered to be green solvent, and recently it gained much attention [35]. E-M algorithm showed that LC_50_ by inhalation is 1186 ppm and LC_50_ towards fish is 756.6 mg dm^−3^ and biodegradability half-life is 7.8 days. These values suggest that this compound is not very toxic and it undergoes biodegradation.

Chloropropane, chlorobutane, and chloropentane are characterized by mean predicted missing value of inhalation LC_50_ = 12572 ppm, which is in accordance with available values for cis-1,2-dichloroethene (13,700 ppm) and 1,1-dichloroethane (13,000 ppm). The completed values show that they are slightly less toxic than the mean of the entire dataset. Mean value of LC_50_ towards fish is 82 mg dm^−3^, what shows they can be toxic to fish as the mean value of this parameters equals to 970 mg dm^−3^. Biodegradability half-lifes are predicted to be 18.2 and 10.5 days for 1-chlorobutane and 1-chloropentane, respectively. This value for 1-chloropropane of 30 days is available in the original dataset. What is more, the completed value for 1,1,2,2-tetrachloroethane is equal to 134 days. Chlorinated solvents are not concerned as green solvents and predicted missing values confirm this statement.

To obtain the information about the standard errors bootstrap analysis is performed [36]. The results of analysis are presented in Table 4. Standard errors are small for almost all of the variables. Only those variables, which do not contain data gaps and are not transformed, have the high standard errors.

## 4. Conclusions

E-M algorithm is useful in predicting of organic solvents missing parameters. Algorithm allows for completing 35 values of LD_50_ by oral administration to rodents, 46 values of LC_50_ by inhalation, 57 values of LC_50_ towards fish, 57 values of biodegradability, and 62 values of bioconcentration factors. Some of the solvents that are considered in this study are promising from the Green Chemistry point of view, even though they have not been fully characterized yet.

E-M algorithm can be useful in characterization of other novel, potential green alternatives of other chemicals. It is important for the characterization of chemicals for their rapid screening.

## Figures and Tables

**Table 1:** Basic statistics of the dataset.

Variable	Mean	Std. Dev.	Variance	Minimum	Maximum	*N*	*N* Missing
Melting point (°C)	−43.901	48.728	2374.453	−140	49.52	152	3
Boiling point (°C)	142.385	68.626	4709.488	20	323	155	0
Density (g cm^−3^)	0.952	0.214	0.046	0.62	1.68	155	0
Water solubility (mg dm^−3^)	116,796.63	244,339.68	5.97 × 10^10^	0.000927	1,000,000	155	0
Vapor pressure (Pa)	11,901.626	28,631.781	8.2 × 10^8^	0	241,900	155	0
Henry law constant (Pa m^3^ mol^−1^)	60,736.714	267,946.02	7.18 ×1 0^10^	8.03×10^−6^	2,219,017	153	2
log K_OW_	2.229	2.352	5.531	−2.32	8.73	155	0
log K_OA_	4.434	1.999	3.995	1.451	12.101	152	3
Oral LD_50_ (mg kg^−1^)	3667.383	4658.48	21,701,436	5	31,500	120	35
Inhalation LC_50_ (ppm)	10,532.284	18,252.957	3.33 × 10^8^	34	123,000	109	46
Fish LC_50_ (mg dm^−3^)	970.096	2813.093	7,913,490	0.1	16,700	98	57
BOD t_1/2_ [days]	55.360	127.192	16,178	1	800	93	62
log BCF	1.154	1.016	1.032	−1.63	4.7	151	4

LD_50_: lethal dose administered orally to rodents that kills half of population; LC_50_: toxicity towards rodents via inhalation exposure pathway; Std. Dev.: Standard Deviation; K_ow_: octanol-water partitioning coefficient; K_OA_: octanol-air partitioning coefficient; BOD: biodegradation half-life; BCF: bioconcentration factor.

**Table 2:** The results of data treatment with principal component analysis. Dark red is for very negative values, yellow is for neutral values and green stands for positive values.

	Comp. 1	Comp. 2	Comp. 3
Melting point	−0.4448	−0.1465	−0.0451
Boiling point	−0.4963	−0.0883	0.0598
Density	−0.0607	−0.0709	−0.4854
Water solubility	0.1150	−0.3744	0.0708
Vapor pressure	0.3478	0.0295	−0.0888
log Henry law const	0.1247	0.5103	0.0289
log K_OW_	−0.2462	0.4340	0.1635
log K_OA_	−0.4352	−0.1795	0.1165
log Oral LD_50_	−0.0528	0.0933	0.5678
log Inhalation LC_50_	0.2287	0.0600	0.4662
log fish LC_50_	0.1673	−0.2642	0.2638
log BOD t_1/2_	0.0848	0.2915	−0.3085
log BCF	−0.2492	0.4202	0.0174

**Table 3:** Input and completed values (shaded) for solvents that were not fully characterized. Green color indicate predicted values.

	Solvent	CAS Number	Oral LD_50_ (mg kg^−1^)	Inhalation LC_50_ (ppm)	Fish LC_50_ (mg dm^−3^)	BOD t_1/2_ (days)	log BCF
1	Cyclopentane	287-92-3	11,400	57,377	100	10.6	1.61
2	Octane	111-65-9	7930	25,260	100	13.7	3.289
3	Nonane	111-84-2	218	3200	6.5	16.4	2.651
4	Decane	124-18-5	5000	1369	500	40.0	2.158
5	Tridecane	629-50-5	5000	41	0.9	18.2	2.979
6	Tetradecane	629-59-4	15,000	5001	1000	38.7	3.036
7	Pentadecane	629-62-9	5000	5001	100.1	39.6	2.34
8	1-pentene	109-67-1	3197	21,800	90.7	17.0	1.349
9	1-hexene	646-04-8	10,000	32,000	5.6	10.7	1.91
10	1-heptene	592-76-7	5000	27,986	175	12.6	2.372
11	1-octene	111-66-0	10,000	8500	6.8	9.3	2.819
12	1-nonene	124-11-8	4390	7116	5.0	9.6	3.266
13	Pentanol	71-41-0	2200	6119	370	4	0.463
14	oleic alcohol	143-28-2	9604	13,049	46.7	9.0	2.623
15	1,3-di-iso-propoxy-2-propanol	13021-54-0	1267	2725	33.5	1.6	0.5
16	1,3-dimethoxypropan-2-ol		1393	3794	104.7	2.5	0.5
17	1,3-di-*n*-butoxy-2-propanol		1130	885	69.4	2.1	0.603
18	1-ethoxy-3-iso-propoxy-2-propanol		1256	1889	377.7	4.3	0.5
19	1-methoxy-3-(propan-2-yloxy)propan-2-ol		1498	2945	160.8	2.1	0.5
20	1-*n*-butoxy-3-ethoxy-2-propanol		2220	2347	232.2	1.8	0.5
21	1-*n*-butoxy-3-iso-propoxy-2-propanol		3047	4273	188.0	1.5	0.168
22	1-*n*-butoxy-3-methoxy-2-propanol		1883	2582	197.6	2.1	0.5
23	1-*tert*-butoxy-3-ethoxy-2-propanol		2568	4601	97.1	1.3	0.5
24	1-*tert*-butoxy-3-methoxy-2-propanol		1477	3305	35.3	1.4	0.5
25	3-butoxypropane-1,2-diol		3875	2818	203.4	1.5	0.5
26	3-ethoxypropane-1,2-diol		2538	2663	186.2	1.9	0.5
27	3-methoxypropane-1,2-diol		2081	1985	272.4	2.6	0.5
28	3-*n*-butoxy-1-*tert*-butoxy-2-propanol		5660	5167	51.7	1.4	0.517
29	Isopropylidene glycerol	100-79-8	7000	167,197	16,700	1.3	0.125
30	Methoxycyclopentane	5614-37-9	1500	5250	34.9	6.6	0.721
31	Benzyl ethyl ether	539-30-0	2428	2625	38.6	6.6	1.374
32	1,2,3-trimethoxypropane		1305	2815	135.8	5.3	0.5
33	1,2,3-*tri*-*n*-butoxypropane		4390	5001	261.2	4.5	2.276
34	2-methylfuran		1965	9352	94.3	16.0	0.725
35	2-methyltetrahydrofuran		4500	24,083	319.6	6.4	0.343
36	3-*n*-butoxy-1-*tert*-butoxy-2-methoxypropane		2392	1656	95.4	2.9	1.094
37	Isosorbide dimethyl ether		1545	18,269	213.8	4.9	0.5
38	Dioxolane	646-06-0	2833	3,7363	31.0	6.1	0.149
39	Benzaldehyde	100-52-7	1300	1304	1.07	10	1.1
40	gamma-valerolactone	108-29-2	2800	1186	756.6	7.8	0.5
41	Dihydrolevoglucosenone		2021	2916	59.3	4.4	0.5
42	1,8-cineole	470-82-6	2480	1000	102	26.4	1.41
43	3-carene	13466-78-9	4800	8800	17.9	28	2.673
44	Neryl acetate	141-12-8	4550	5001	41.7	8.7	2.365
45	Propionic acid	79-19-4	3500	5422	51	1	0
46	Ethyl formate		1850	9800	276.6	15	0.5
47	Butyl levulinate	2052-15-5	5000	5001	26.3	3.3	0.278
48	Ethyl levulinate	539-88-8	5000	4735	121.3	3.3	0.5
49	Glycerol triacetate	102-76-1	3000	5001	72.5	2.2	0.5
50	Methyl caprylate	111-11-5	10,800	9987	95	7.0	1.856
51	Methyl lactate	27871-49-4	5000	1350	828.6	11.8	0.5
52	Methyl levulinate	624-45-3	2051	2888	92.7	3.4	0.5
53	Methyl linoleate	112-63-0	3977	5001	4.5	20.4	3.051
54	Isopropyl myristate	110-27-0	8348	11,207	8.4	10.7	3.07
55	Methyl oleate	112-62-9	2000	5001	6.1	18.9	2.694
56	Methyl palmitate	112-39-0	4786	5001	1.8	9.4	2.789
57	Isopropyl palmitate	142-91-6	17,781	45,414	50.3	13.0	1.725
58	Methyl stearate	112-61-8	5237	5001	2.8	10.4	1.46
59	Tributyl 2-acetylcitrate	77-90-7	31,500	226,174	60	14	1.6
60	Benzyl benzoate	120-51-4	1700	665	6.2	5.3	2.357
61	*cis*-1,2-dichloroethene	156-59-2	1393	13,700	54.2	180	1.18
62	1,1-dichloroethane	75-34-3	725	13,000	100.0	154	1.24
63	1,1,1,2-tetrachloroethane	630-20-6	670	2100	20	134.0	1.559
64	1-chloropropane	540-54-5	2000	14,034	117.8	30	0.763
65	1-chlorobutane	109-69-3	2670	11,879	101.2	18.2	1.333
66	1-chloropentane	543-59-9	3379	11,804	27.8	10.5	1.402
67	Dimethyl sulphide	75-18-3	535	5156	87.1	10.7	0.561
68	Dimethyl sulfoxide	67-68-5	2758	4291	36.9	1.6	0.349
69	Diethylamine	109-89-7	540	4000	218.5	5.0	0.21
70	2-pyrrolidone	616-45-5	2030	1083	152.5	2.5	0.5

**Table 4:** Standard errors of predictions calculated with bootstrap.

Variable	Mean	Mean Error
Melting point (°C)	−43.1378	−0.0056
Boiling point (°C)	142.3852	−0.4245
Density (g cm^−3^)	0.9521	−0.0005
Water solubility (mg dm^−3^)	116,796.6328	−279.7044
vapor pressure (Pa)	11,901.6258	403.5914
Henry law constant (Pa m^3^ mol^−1^)	2.4677	0.0955
log K_OW_	2.2285	0.0125
log K_OA_	4.4644	−0.0269
Oral LD_50_ (mg kg^−1^)	7.6122	−0.0093
Inhalation LC_50_ (ppm)	8.2734	0.0014
Fish LC_50_ (mg dm^−3^)	4.2248	−0.0069
BOD t_1/2_ (days)	2.2431	0.0065
log BCF	1.1450	0.0076

## Supplementary Materials

The following are available online. E-M algorithm description.
