# Supplementary material for: Expectation-Maximization Model for Substitution of Missing Values Characterizing Greenness of Organic Solvents
Source: Molecules. 2018 May 28;23(6):1292. doi: 10.3390/molecules23061292 (PMC6100055; doi:10.3390/molecules23061292)
Supplement: Supplementary file 1 [file molecules-23-01292-s001.pdf]

Supplementary material

Article

Expectation-Maximization model for substitution of missing values characterizing greenness of organic solvents

Gabriela Łuczyńska [1,2] Francisco Pena-Pereira [3], Marek Tobiszewski [4] and Jacek Namieśnik* [4]

[1] Division of Applied Mathematics and Probability, Institute of Mathematics, Faculty of Mathematics, University of Gdansk, 8 J. Bażyńskiego St., 80-309 Gdańsk, Poland; gabluczy@student.pg.edu.pl
[2] Department of Nonlinear Analysis and Statistics, Faculty of Applied Mathematics, Gdańsk University of Technology (GUT), 11/12 G. Narutowicza St., 80-233 Gdańsk, Poland
[3] Department of Analytical and Food Chemistry, Faculty of Chemistry, University of Vigo, Campus As Lagoas-Marcosende s/n, 36310 Vigo, Spain; fjpena@uvigo.es
[4] Department of Analytical Chemistry, Chemical Faculty, Gdańsk University of Technology (GUT), 11/12 G. Narutowicza St., 80-233 Gdańsk, Poland; marektobiszewski@wp.pl
* Correspondence: jacek.namiesnik@pg.edu.pl or chemanal@pg.edu.pl

E-M algorithm description

To complete the data we use E-M algorithm. This algorithm consists of two steps: an Expectation step or the E-step and a Maximization step or the M-step.

We observe a data vector $\boldsymbol{y}$. Let $\boldsymbol{Y}$ be the random vector corresponding to the data $\boldsymbol{y}$. Let $\boldsymbol{\Theta}$ be a parameter space and let $\boldsymbol{Y}$ has the probability distribution function $g(\boldsymbol{y};\boldsymbol{\Psi})$ where $\boldsymbol{\Psi}$ is a vector of unknown parameters from $\boldsymbol{\Theta}$.

Let $\boldsymbol{X}$ be a random vector corresponding to a complete-data vector $\boldsymbol{x}$. Denote the probability distribution function of $\boldsymbol{X}$ by $g_c(\boldsymbol{x};\boldsymbol{\Psi})$. Let $\boldsymbol{A}$ and $\boldsymbol{B}$ be two samples spaces in which we observe the data $\boldsymbol{x}$ and $\boldsymbol{y}$ respectively. We don't observe the complete vector $\boldsymbol{x}$ in $\boldsymbol{A}$ but only the incomplete vector $\boldsymbol{y}=\boldsymbol{y}(\boldsymbol{x})$ in $\boldsymbol{B}$. Therefore we have a many-to-one mapping from $\boldsymbol{A}$ to $\boldsymbol{B}$ and due to the disintegration theorem [28]

$$g(\boldsymbol{y};\boldsymbol{\Psi}) = \int_{\boldsymbol{A}(\boldsymbol{y})} g_c(\boldsymbol{x};\boldsymbol{\Psi})\,dx, \tag{1}$$

where $\boldsymbol{A}(\boldsymbol{y})$ is a subset of $\boldsymbol{A}$ determined by the equation $\boldsymbol{y}=\boldsymbol{y}(\boldsymbol{x})$. The likelihood function for $\boldsymbol{\Psi}$ formed from the observed data $\boldsymbol{y}$ is given by

$$L(\boldsymbol{\Psi}) = g(\boldsymbol{y};\boldsymbol{\Psi}). \tag{2}$$

An estimate $\widehat{\boldsymbol{\Psi}}$ of $\boldsymbol{\Psi}$ can be obtained by solving the log likelihood equation

$$\frac{\partial \log L(\boldsymbol{\Psi})}{\partial \boldsymbol{\Psi}} = 0, \tag{3}$$

where log is the natural logarithm function. The E-M algorithm approaches the problem of solving the incomplete-data log likelihood equation (3) indirectly by proceeding iteratively in terms of complete-data log likelihood function $\log L_c(\boldsymbol{\Psi})$, where

$$L_c(\boldsymbol{\Psi}) = g_c(\boldsymbol{x};\boldsymbol{\Psi}). \tag{4}$$

Since it is unobservable, it is replaced by its conditional expected value given $\boldsymbol{y}$, using the current fit for $\boldsymbol{\Psi}$.

Let $\mathbf{\Psi}^{(0)}$ be some initial value for $\mathbf{\Psi}$. Then on the first iteration in the E-step we calculate

$$Q\left(\mathbf{\Psi}, \mathbf{\Psi}^{(0)}\right) \coloneqq E_{\mathbf{\Psi}^{(0)}}(\log L_c(\mathbf{\Psi})|\boldsymbol{y}). \tag{5}$$

After that, in the M-step we maximize $Q\left(\mathbf{\Psi}, \mathbf{\Psi}^{(0)}\right)$ with respect to $\mathbf{\Psi}$ over the parameter space $\mathbf{\Theta}$. In other words we choose such $\mathbf{\Psi}^{(1)}$ that

$$\forall_{\mathbf{\Psi}\in\mathbf{\Theta}}\, Q\left(\mathbf{\Psi}^{(1)}, \mathbf{\Psi}^{(0)}\right) \geq Q\left(\mathbf{\Psi}, \mathbf{\Psi}^{(0)}\right). \tag{6}$$

Note that this choice doesn't have to be unique.

Thereafter steps are perform again with value $\mathbf{\Psi}^{(1)}$ in place of $\mathbf{\Psi}^{(0)}$. The $(k+1)$th iteration of the E-M algorithm requires in the E-step the calculation of

$$Q\left(\mathbf{\Psi}, \mathbf{\Psi}^{(k)}\right) \coloneqq E_{\mathbf{\Psi}^{(k)}}(\log L_c(\mathbf{\Psi})|\boldsymbol{y}). \tag{7}$$

and in the M-step the choice of $\mathbf{\Psi}^{(k+1)} \in \mathbf{\Theta}$ which maximizes $Q\left(\mathbf{\Psi}, \mathbf{\Psi}^{(k)}\right)$ with respect to $\mathbf{\Psi} \in \mathbf{\Theta}$, i.e.

$$\forall_{\mathbf{\Psi}\in\mathbf{\Theta}}\, Q\left(\mathbf{\Psi}^{(k+1)}, \mathbf{\Psi}^{(k)}\right) \geq Q\left(\mathbf{\Psi}, \mathbf{\Psi}^{(k)}\right). \tag{8}$$

The steps are carried out until the value of

$$L\left(\mathbf{\Psi}^{(k+1)}\right) - L\left(\mathbf{\Psi}^{(k)}\right) \tag{9}$$

is smaller than arbitrarily amount in case of convergence of the sequence of likelihood values $\left(L\left(\mathbf{\Psi}^{(k)}\right)\right)_k$. It has been shown in [29] that this sequence is nondecreasing. Therefore it is convergent, if it is bounded above.
